# Trinuclear Oxo-Titanium Clusters: Synthesis, Structure, and Photocatalytic Activity

**DOI:** 10.3390/ma12193195

**Published:** 2019-09-29

**Authors:** Maciej Janek, Tadeusz M. Muzioł, Piotr Piszczek

**Affiliations:** Faculty of Chemistry, Nicolaus Copernicus University in Toruń, Gagarina 7, 87-100 Toruń, Poland; maciejjanekk@gmail.com (M.J.); tadeuszmuziol@wp.pl (T.M.M.)

**Keywords:** Titanium (IV) oxo-clusters, structure, photocatalytic activity, bandgap modification, DFT calculations, polymer/inorganic composite systems

## Abstract

The interest in titanium (IV) oxo-complexes is due to their potential application in photodegradation processes and environmental pollutants reduction. Titanium (IV) oxo-complexes (TOCs) of the general formula [Ti_3_O(O^i^Pr)_8_(OOCR’)_2_] (R’ = -C_13_H_9_ (**1**), -*p*-PhCl (**2**), *-m-*PhNO_2_ (**3**), -C_4_H_7_ (**4**)) were synthesized and structurally characterized. The use of the different carboxylate ligands allowed modulating the optical band gaps of the produced microcrystals, which were measured via diffuse reflectance ultraviolet and visible spectroscopy (UV-Vis-DRS) and calculated using the density functional theory (DFT) method. The dispersion of TOCs (**1**–**3**) in the poly (methyl methacrylate) matrix (PMMA) led to the formation of polymer/TOCs composites, which in the next stage of our works have been applied in the photocatalytic activity estimation of synthesized trinuclear Ti(IV) oxo-complexes. Studies of the photocatalytic degradation of methylene blue (MB) induced by UV irradiation exhibit that the PMMA-TOCs composite containing (**1**) oxo-clusters is the most active, followed by the system containing the complex (**3**).

## 1. Introduction

Studies on multinuclear titanium (IV) oxo-complexes (TOCs) are interesting due to the fact that their discrete molecular structure provide insight into correlations between the composition and photophysical properties of these compounds [1,2]. Analyses of property changes in TOCs are especially important. For example, their photoinduced activity towards organic substances degradation, resulting from the similarity of these materials to TiO_2_ [3]. In many cases, the presence of organic ligands (-OR, -O_2_CR’) in the structure of oxo-clusters serve both as multidentate stabilizers of {Ti_a_O_b_} core (e.g. carboxylates, phosphonates), but also as a functionality provider that allows for the acquisition of the unique properties of new materials [4,5]. The knowledge gained based on the results of this investigation enable the rational design and the fabrication of TOCs-based materials as photocatalytic systems and their application as a molecular tool to enhance and modulate photophysical properties of the composite materials.

According to earlier reports, the unfunctionalized TOCs are characterized by a wide band gap, e.g., 3.60 eV for [Ti_8_O_8_(OH)_4_(CO_2_)_12_] [6] (to compare, the band gap for the rutile is 3.03 and 3.23 eV for anatase [7]). The efficient band gap modulation arising from the core-ligands interaction is possible as a result of introduction of photoactive functionalities to the structure of TOCs, which was confirmed by numerous investigations. An excellent example has been displayed in work of Liu et al. [8], where various carboxylate ligands were introduced to labile coordination sides of hexanuclear [Ti_6_O_4_(O^i^Pr)_10_(O_3_P-Phen)_2_(OAc)_2_] complex. Authors were able to modulate band gap values of series of analogous compounds in 3.6–3.0 eV range only by the change of organic functionalities. The more subtle modulation method of band gap values consists in changes of the skeleton composition, while functionalized ligands are maintained. Cui et al. have shown this approach in case of 4-chlorosalicylate stabilized titanium-oxo complexes with different cluster architecture [9]. The resulting optical band gaps changed by a small value, but adequate photo-response differed as oxo-titanium skeleton played a big role in photoactive behaviour of TOCs. The last important approach to alter the characteristic of TOCs, namely the heteroatomic doping of oxo-core, is of great importance. The metal atom can be incorporated into oxo-core architecture and ligands with functionalities possessing lone electron pairs may be used to coordinate to the heteroatomic centre. Additional states introduced by these heteroatoms greatly alter electronic structure of TOCs and their photoinduced behaviour [10]. Our previous works on synthesis and structural characterization of [Ti_4_O_2_(O^i^Bu)_10_(O_2_CR’)_2_] (R = -C_13_H_9_, -*m-*PhCl, -*p-*PhNH_2_ and -*m-*PhNO_2_) oxo-complexes revealed the possibility of modulation of the band gap value of material by anchoring of the different carboxylate ligands to the {Ti_4_O_2_} skeleton [11,12]. This type of compound was used in the fabrication of polymer/TOCs composites (PMMA/TOCs, PMMA = poly (methyl methacrylate), TOCs = {Ti_4_O_2_} clusters), which exhibited promising photocatalytic activity in UV photoinduced degradation processes of methylene blue (MB). Such factors as: (a) low band gap value, (b) *n*-doped character of the compound, (c) ability to generate Ti(III) states upon irradiation and(d) dispersion of titanium oxo-complex nanocrystals in polymer matrix, had a big impact on photocatalytic properties of materials. Considering the results of carried out works, it should be noted that further studies on synthesis and properties of the novel polymer/TOCs-based composite materials, requires the precise analysis of cores size tailoring and their architecture.

Continuing earlier works we have focused on the synthesis of trinuclear Ti (IV) oxo-complexes, characterization of their structure and the estimation of their photocatalytic activity (analysed in the form of polymer/TOCs system). The synthesis and structure of trinuclear Ti (IV) oxo-clusters have been earlier described by Boyle et al. [13], and Mijatovic et al. [14], and Czakler et al. [15]. Simultaneously, discussing the stepwise assembly processes of larger {Ti_a_O_b_} cores Schubert suggested that oxo-complex with the {Ti_3_O} core is the basic unit, which in proper conditions leads to the formation of [Ti_a_O_b_(OR)_c_(O_2_CR’)_4a-2b-c_] clusters [16]. In our research, we have decided to carry out syntheses of [Ti_3_O(O^i^Pr)_8_(O_2_CR’)_2_] systems using the novel group of organic acids, i.e. HOOCR’, R’ = -C_13_H_9_, -p-PhCl, and -m-PhNO_2_, -C_4_H_7_, which were not studied yet in terms of this topic. The aim of our studies was to determine the influence of the organic acid type on the core {Ti_3_O} structure, on the size of the energy band gap, as well as on the photocatalytic activity of isolated trinuclear Ti(IV) oxo-clusters. The estimation of this effect is especially important for the future application of polymer materials enriched with Ti (IV) oxo-complexes, as the photocatalytic systems used in the biological/organic pollutants degradation.

## 2. Materials and Methods

### 2.1. Materials

Titanium(IV) isopropoxide (Aldrich, St. Louis, MO, USA), 4-chlorobenzoic acid (Aldrich, St. Louis, MO, USA), 3-nitrobenzoic acid (Aldrich, St. Louis, MO, USA), 3,3-dimethylacrylic acid Aldrich, St. Louis, MO, USA), and 9-fluorenecarboxylic acid (Organic Acro, Geel, Belgium) were purchased commercially and were used without further purification. Tetrahydrofuran (THF) was distilled before using. Standard Schlenk techniques were used for synthesis under an inert gas atmosphere.

### 2.2. Synthesis

The synthesis of [Ti_3_O(O^i^Pr)_8_(O_2_CC_13_H_9_)_2_] (**1**). 0.184 g of 9-fluorenecarboxylic acid (0.875 mmol) was added to the solution of 1 ml titanium (IV) isopropoxide (3.5 mmol) in 2 ml of THF/^i^PrOH (1:1). Reactants underwent rapid reaction leading to clear brownish solution. The solution was left for crystallization. Crystalline product was collected after 3 days. The yield basing on acid: 74% (0.33 g). Anal. Calc. for C_50_H_74_O_13_Ti_3_: C, 58.49; H, 7.26. Found: C, 58.76; H, 7.16.

The synthesis of [Ti_3_O(O^i^Pr)_8_(O_2_CC_6_H_4_Cl)_2_] (**2**). 0.137 g of 4-chlorobenzoic acid (0.875 mmol) was added to the solution of 1 ml titanium(IV) isopropoxide (3.5 mmol) in 2 ml of THF/^i^PrOH (1:1), leading to a colourless solution. The solution was left for crystallization. Evaporation under inert gas atmosphere led to crystals suitable for X-ray diffraction experiment. The yield basing on acid: 82% (0.34 g). Anal. Calc. for C_38_H_64_O_13_Cl_2_Ti_3_: C, 48.38; H, 6.84. Found: C, 49.12; H, 6.64.

The synthesis of [Ti_3_O(O^i^Pr)_8_(O_2_CC_6_H_4_NO_2_)_2_] (**3**). 0.146 g of 3-nitrobenzoic acid (0.875 mmol) was added to the solution of 1 ml titanium (IV) isopropoxide (3.5 mmol) in 2 ml of THF/^i^PrOH (1:1), leading to a weak yellow solution. The solution was left for crystallization. Slow evaporation under an inert gas atmosphere led to crystals suitable for X-ray diffraction experiment. The yield basing on acid: 67% (0.28 g). Anal. Calc. for C_38_H_64_O_17_N_2_Ti_3_: C, 47.32; H, 6.69; N, 2.90. Found: 58.25; H, 6.94; N, 2.58.

The synthesis of [Ti_3_O(O^i^Pr)_8_(O_2_CC_4_H_7_)_2_] (**4**). 0.088 g of 3,3 dimethylacrylic acid (0.875 mmol) was added to the solution of 1 ml titanium(IV) isopropoxide (3.5 mmol) in 2 ml of THF/^i^PrOH (1:1), leading to a colourless solution, which was left for crystallization. Slow evaporation under an inert gas atmosphere led to crystals. The yield basing on acid: 78% (0.28 g). Anal. Calc. for C_34_H_70_O_13_Ti_3_: C, 58.49; H, 7.26. Found: C, 58.76; H, 7.16.

### 2.3. Analytical Procedures

The vibrational spectra of synthesized compounds were recorded using the Perkin Elmer Spectrum 2000 FT-IR spectrometer (400–4000 cm^−1^ range, KBr pellets, Spectrum2000, PerkinElmer Inc., Waltham, MA**,** USA) and the RamanMicro 200 Perkin Elmer spectrometer (PerkinElmer Inc., Waltham, MA, USA) (l = 785 nm). The solid state optical diffuse-reflection experiment was carried out on the Jasco V-750 Spectrophotometer (Jasco Corporation, Tokyo, Japan) equipped with an integrating sphere for diffuse reflectance spectroscopy. Spectralon® was used as the DRS refference sample. Elemental analyses were performed on Elemental Analyser vario Macro CHN (Elementar Analysensysteme GmbH, Langenselbold, Germany). The dispersion of nano/microcrystals in polymer matrix was estimated using a Quanta field emission scanning electron microscope (FESEM, Quanta 3D FEG, Huston, TX, USA).

### 2.4. X-Ray Crystalography Study

For single crystals, the diffraction data of (**2**) and (**3**) were collected using BL14.3 beamline (Helmholtz Zentrum Berlin, Bessy II), radiation λ = 0.89429 Å, at liquid nitrogen temperature, whereas for (**1**) the diffraction experiment was performed at room temperature, using Oxford Sapphire CCD diffractometer, MoKα radiation λ = 0.71073 Å. The data were processed using CrysAlis [17], *xdsapp* [18], XDS [19], and the numerical absorption correction was applied for all crystals. The structures of all complexes were solved by the direct methods and refined with full-matrix least-squares procedure on F2 (SHELX-97 [20]). All heavy atoms were refined with anisotropic displacement parameters. The positions of hydrogen atoms were assigned at calculated positions with thermal displacement parameters fixed to a value of 20% or 50% higher than those of the corresponding carbon atoms. For (**2**) some constraints (ISOR for C15 atom) were applied. In (**2**) the alternate positions were found only for an aliphatic chain of O11 -O^i^Pr. All figures were prepared in DIAMOND [21] and ORTEP-3 [22]. The results of the data collections and refinement are summarized in Table 1.

### 2.5. Preparation and Photocatalytic Activity Studies of Composites

In order to obtain composite (PMMA/TOCs) foils with 20 *wt.%* of trinuclear TOCs (**1**) (**3**), the following procedure was applied: (a) 1 g of poly(methyl methacrylate) (PMMA) was dissolved in THF (10 ml); (b) a mixture of 0.25 g of TOCs (**1**–**3**) in 2 ml of THF was added to the clear stirring solution (a) and stirred for 30 min; (c) the resulting solution was poured into a glass Petri dish and left for 2 days for solvent evaporation; (d) the composite foil was collected and prepared for a photocatalytic activity experiments. In our works we have focused in studies of (**1**–**3**) complexes of carboxylate ligands similar to these which are used in our earlier photocatalytic experiments with the use of {Ti_4_O_2_} cores [12].

8 × 8 mm composite foil samples were prepared for every photocatalytic activity test, put into the bottom of quartz cuvettes and covered with 3 ml of methylene blue solution (c = 1.0 × 10^−5^ M). Samples in cuvettes were irradiated with UV light source (18 W, range of 340–410 nm with maximum at 365 nm), being located 20 cm above the irradiation system. Absorbance values at 664 nm were measured every 24 hours for every sample.

In order to evaluate MB degradation kinetics, the Langmuir–Hinshelwood reaction mechanism was assumed [23]. For low concentrations *c* the relation simplifies as follows:

*r* = −*dc*/*dt* = *k*_deg_*Kc*/(1 + *Kc*) ≈ *k*_deg_*Kc* = *k*_obs_*c*

where *c* is a methylene blue concentration at a given time *t*, *k*_deg_ is the rate constant of methylene blue, decomposition on the foil surface, *K* describes the reactant adsorption–desorption process, and *k*_obs_ is a pseudo-first order observed rate constant.

The slope of the following relation gives the apparent pseudo-first order rate constant:

*-ln(c/c_0_) = k_obs_t*

where *c_0_* is an initial concentration of MB, *c* is a MB concentration at a given time *t*, and *k*_obs_ is a pseudo-first order observed rate constant.

The MB decolorization percent was calculated using the following formula:

*% MB decolorization* = (*c_0_ – c_t_/c_0_*)·100

where *c_0_* is an initial concentration of MB, *c* is a MB concentration at a given time *t* [24].

### 2.6. The Computational Details

The crystal structures were used as a starting point of the geometry optimization stage, with exception of isopropyl groups, which were substituted with methyl groups to reduce the cost of calculations. Gaussian09 packages with B3LYP functional and 6-31G (d) basis set was used for DFT calculations [25]. The converged structures were confirmed as true local minima at the potential energy surface by no imaginary frequencies criterion. DOS plots were made with the help of the GaussSum 3.0 software [26].

## 3. Results

### 3.1. Synthesis and Structures of [Ti_3_O(O^i^Pr)_8_(O_2_CR’)_2_] clusters

The (**1**–**4**) oxo-complexes were synthesized in the direct reaction of titanium (IV) isopropoxide and organic acids in 4:1 alkoxide/acid molar ratio in 1:1 THF/^i^PrOHmixture as a solvent, using standard *Schlenk* techniques under an argon atmosphere and room temperature (RT). The following organic acids were used in our experiments: 9 fluorenecoarboxylic acid (HOOCC_13_H_9_), 4-chlorobenzoic acid (HOOC-*p*-PhCl), 3-nitrobenzoic acid (HOOC-*m*-PhNO_2_), and 3,3-dimethylacrylic acid (HOOCC_4_H_7_). The slow evaporation of the reaction liquors under an inert gas atmosphere (3–5 days), led to the isolation of crystalline products, which depending to the used organic acid were labelled as(**1**) (R’ = -C_13_H_9_), (**2**) (R’ = *-p*-PhCl), (**3**) (R’ =-*m*-PhNO_2_), and(**4**) (R’ =-C_4_H_7_), respectively. Single crystal X-ray diffraction studies revealed that quality of only (**2**) and (**3**) crystals were adequate to solve and refine their structure. The quality of the obtained (**1**) and (**4**) reaction products did not allow for determining of their structure by X-ray diffraction method and required the use of spectroscopic methods for this purpose.

**Figure 1:**
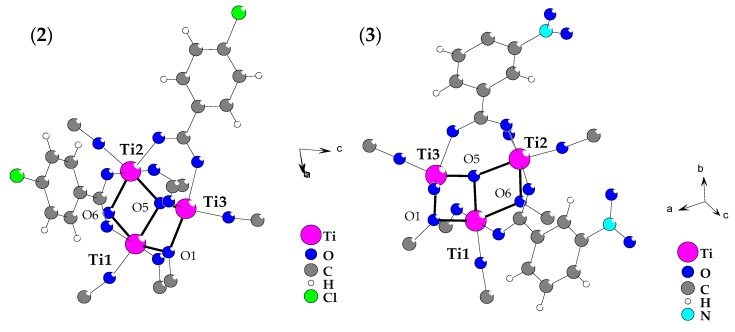
Structure of {Ti_3_O} core, which was found in [Ti_3_O(OR)_8_(O_2_CR’)_2_] (R = ^i^Pr, R’ = PhCl (**2**), PhNO_2_ (**3**) complexes (crystallographic ball-stick scheme). For clarity, the terminal alkoxide groups are omitted.

Analysis of diffraction data collected for (**2**) and (**3**) proved the formation[Ti_3_O(O^i^Pr)_8_(O_2_CR’)_2_] complexes (R’ = -*p*-PhCl (**2**), -*m*-PhNO_2_ (**3**)), which central part consists of µ_3_-O bridges linking by three Ti(IV) atoms in distorted trigonal planar {Ti_3_O} core. Two (µ_2_-O^i^Pr) bridges, two *syn-syn* carboxylate groups, and eight terminal alkoxide ligands stabilize trinuclear titanium oxo-core, as it is shown in Figure 1. The selected bonds lengths and angles, which were found in structures of (**2**) and (**3**) are listed in Table 2. The coordination spheres of three titanium atoms showed that: (a) Ti1 and Ti2 are found in octahedral environment, whereas Ti3 is coordinatively unsaturated with τ_5_ being 0.65 and 0.64 for (**2**) and (**3**), respectively (τ_5_: the parameter defined as (alpha-beta)/60 with alpha and beta being the largest angles in the coordination sphere [27]), (b) their coordination spheres content is different being Ti(μ_3_-O)(μ-OR)_2_(μ-OOCR’)(OR)_2_, Ti(μ_3_-O)(μ-OR)(μ-OOCR’)_2_(OR)_2_ and Ti(μ_3_-O)(μ-OR)(μ-OOCR’)(OR)_2_ for Ti1, Ti2 and Ti3 atoms, respectively. Comparing the structural data of these complexes allows tracing the carboxylate group’s kind influence on the geometry of the {Ti_3_O} core, which may be associated with their photocatalytic activity. According to earlier reports concerning photoactivity of Ti(IV) oxo-clusters, the possible changes of titanium-oxo bridges angles should be noted, which can be responsible for the facilitation of the photocatalytic process [28].

The comparison of Ti-(µ_3_-O) distances and Ti-(µ_3_-O)-Ti angles revealed similarities between geometry of oxo-bridges in the structure of (**2**) and (**3**) complexes. It should be noted that Ti-(µ_3_-O) bonds are much shorter for five-coordinated Ti3 atoms found in heavily distorted trigonal bipyramidal surrounding and the Ti3-O5-Ti2 angle is also significantly bigger than two remaining Ti-O5-Ti angles. The shift of the oxygen atom relative the plane formed by the three atoms of titanium of {Ti_3_-O} core, being 0.36 Å for (**2**) and 0.24 Å for (**3**), is especially interesting. However, this oxo-anion is buried in the hydrophobic cavity between three ^i^Pr groups and is not involved into intermolecular interactions.

### 3.2. Spectral Characterization of Trinuclear Ti(IV) Oxocomplexes

Due to the low crystals quality of (**1**) and (**4**) oxo-clusters, their structures were determined on the basis of IR and Raman spectra analysis. The following spectral ranges were analyzed: (a) below 800 cm^−1^, (b) 1400–1700 cm^−1^, and (c) 900–1050 cm^−1^, where appears the bands derived to stretching and bending vibrations of {Ti_3_O} bridges, carboxylato and alkoxide groups, coordinated to the oxo-core, respectively. The vibrational spectroscopy analysis was assisted by DFT methods. DFT calculations (B3LYP/6-31G(d) level of theory) have been carried out for studied {Ti_3_O} core linked with two carboxylate ligands ((**1**–**4**)) and stabilized by eight alkoxide groups (XYZ atom coordinates of the optimized structures are presented in Tables S1–S4). Results of these works are collected in Table 3.

**Figure 2:**
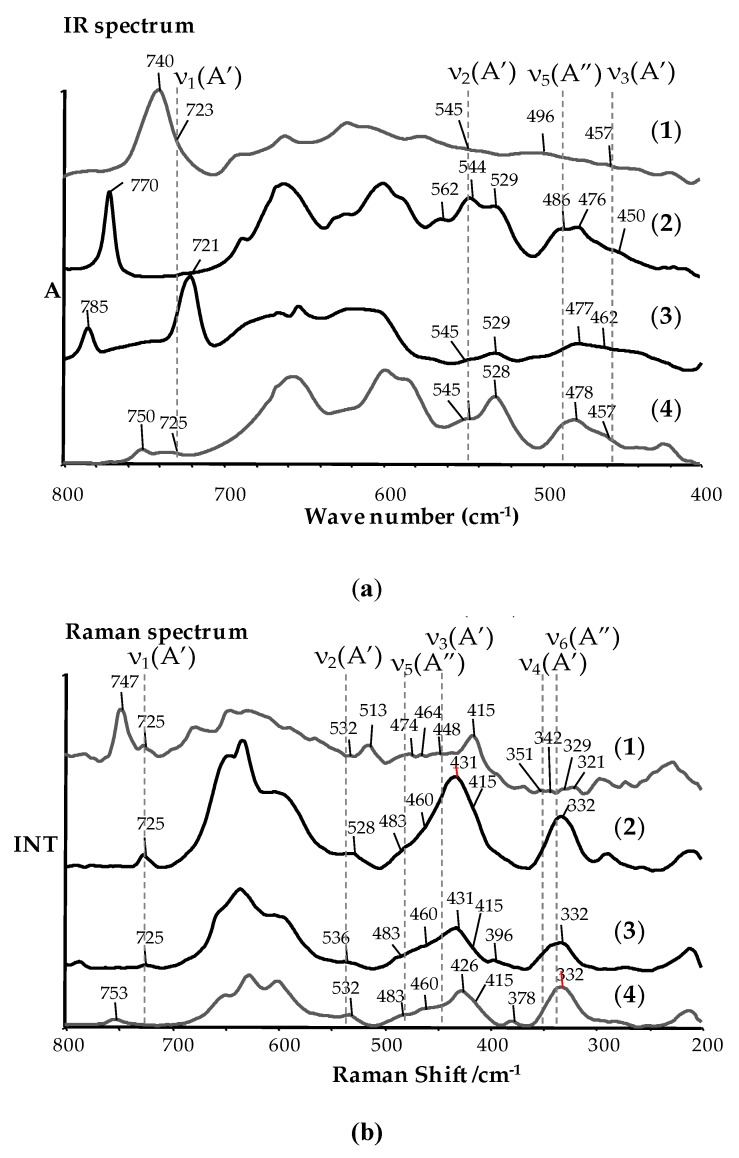
IR (**a**) and Raman (**b**) spectra of (**1**–**4**) complexes, registered in the range of appearance of bands coming from vibrations of {Ti_3_O} bridges.

In our calculations, O^i^Pr ligands were exchanged on the OMe groups for sake of simplicity, moreover the calculations for the [T_i3_O(OMe)_8_(OOCMe)_2_] cluster has been carried out as a reference system. The spectral data for above mentioned regions of infrared (IR) and Raman spectra are presented in Table 4 and Figure 2, while the whole IR and Raman spectra of studied compounds are presented as the supplementary data (Figures S1 and S2). The results of our earlier spectral studies (IR, Raman) of titanium(IV) oxo-complexes (containing the {Ti_a_O_b_} moiety) revealed that the bands of very weak (vw) or middle (m) intensity, which can be assigned to vibrations μ_i_-O bridges (i-2, 3, 4) appears between 300 and 900 cm^−**1**^ [11,12,30,31]. The bands registered at 480–900 cm^−1^ can be assigned to stretching modes of ν(Ti-O) modes, while the bands at 300–480 cm^−1^ to bending δ(Ti-O-Ti) ones.

### 3.3. UV-Vis Absorption Spectra, Band Gap Determination and DOS Plots

**Figure 3:**
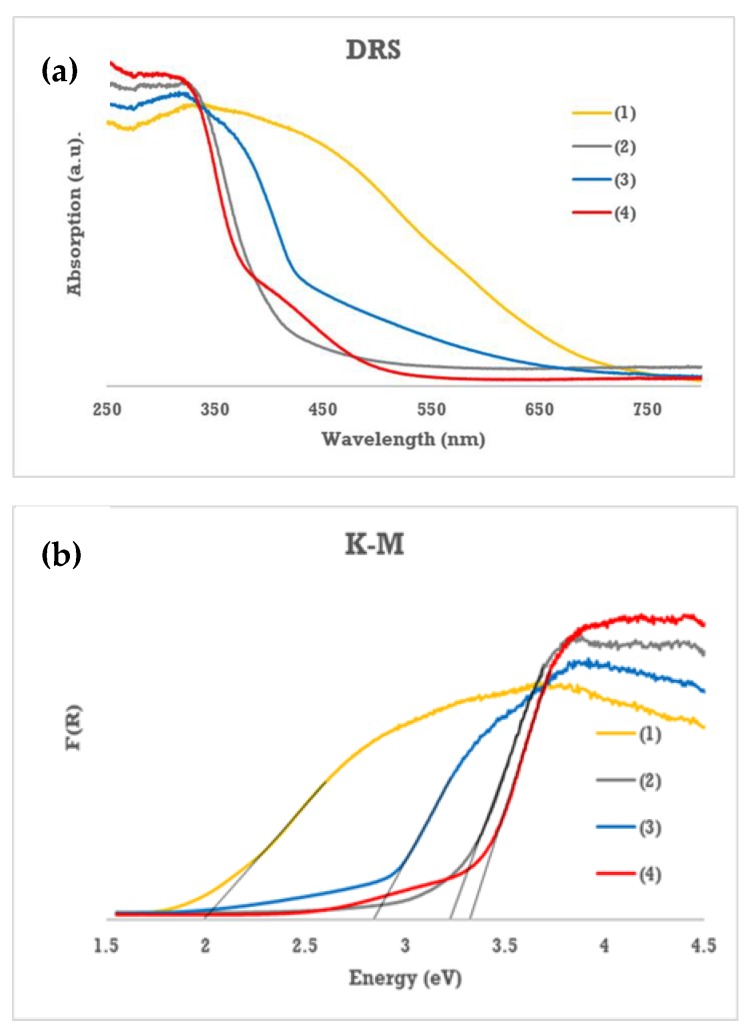
UV-Vis-DRS spectra of (**1**–**4**) complexes (**a**) and Kubelka–Munk function versus light energy plot for the band gap determination (**b**).

The optical band gaps of studied complexes were determined using the diffuse reflectance spectroscopy (UV-Vis-DRS). According to this method, the band gap values have been designated on the basis on the extrapolation of the linear part of Kubelka–Munk (K-M) function versus light energy, i.e. K = f(hν) (K = (1 − R)^2^/2R, R is reflectance) [32,33]. Results of these studies are presented in Figure 3 and Table 5.

**Figure 4:**
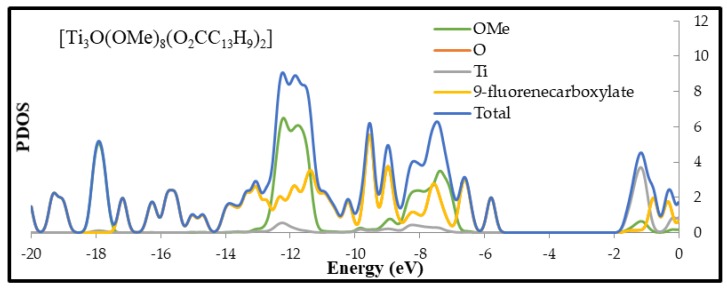

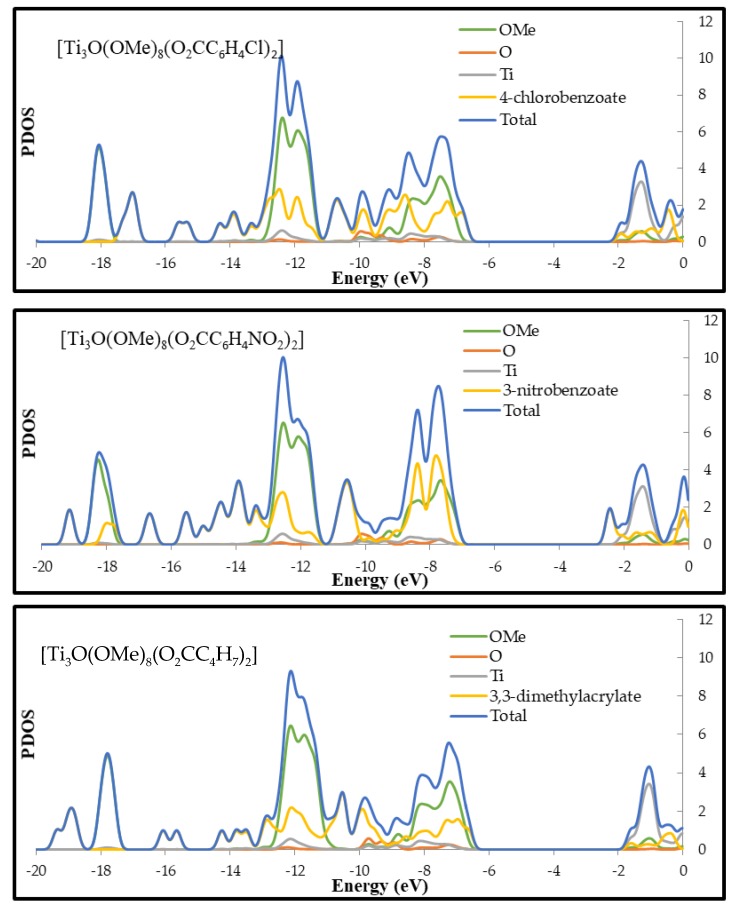
Plots calculated with B3LYP/6-31G(d) level of theory for optimized geometries of [Ti_3_O(OMe)_8_(O_2_CC_13_H_9_)_2_], [Ti_3_O(OMe)_8_(O_2_CC_6_H_4_-Cl)_2_], [Ti_3_O(OMe)_8_(O_2_CC_6_H_4_-NO_2_)_2_], and [Ti_3_O(OMe)_8_(O_2_CC_4_H_7_)_2_] clusters.

According to this data, compounds (**2**) and (**4**) absorption threshold is localized in the UV region of the DRS spectrum and is supported by optically determined band gaps of 3.23 and 3.33 eV respectively. While, in the case of (**3**) and (**1**) complexes these thresholds are shifted towards the Visible part of this spectrum. Corresponding band gaps of these complexes were evaluated as 2.84 and 1.99 eV for (**3**) and (**1**), respectively. The experimentally determined band gap values have been supported by DFT calculations of modeled molecules of (**1**–**4**) clusters (Figure 4 and Table 5). In carried out calculations, the -O^i^Pr ligands have been exchanged on -OMe groups for simplicity. According to data listed in Table 5 we observed the clear differences of the energy gap values determined using experimental and theoretical methods, however this agrees with the tendency of hybrid density functionals to overestimate predicted HOMO–LUMO gaps [34].

Analysis of DFT calculation data presented in Table 5 proved the clear dependence between the band gap value and the type of carboxylate group, which stabilizes the {Ti_3_O} core. The trend of these changes is in good agreement with experimental data.

### 3.4. Estimation of Photocatalytic Activity of Trinuclear Ti(IV) Oxo-Complexes

**Figure 5:**
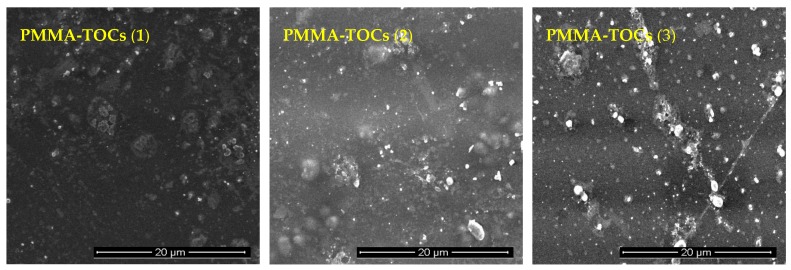
Scanning electron microscopy (SEM) images of PMMA/TOCs composite foils (PMMA = poly(methyl methacrylate); TOCs = (**1**), (**2**), (**3**)) used in photocatalytic experiments.

The photocatalytic activity estimations were carried our using polymer/TOCs composite foils produced by the dispersion of TOCs (**1**–**3**) in the polymer solution (poly(methyl methacrylate) (PMMA)) and slow evaporation of the solvent. Scanning electron microscopy (SEM) confirmed the presence of uniformly dispersed microcrystalline powders of studied oxo-complexes in the composite films of 25–50-μm thick (Figure 5).

**Figure 6:**
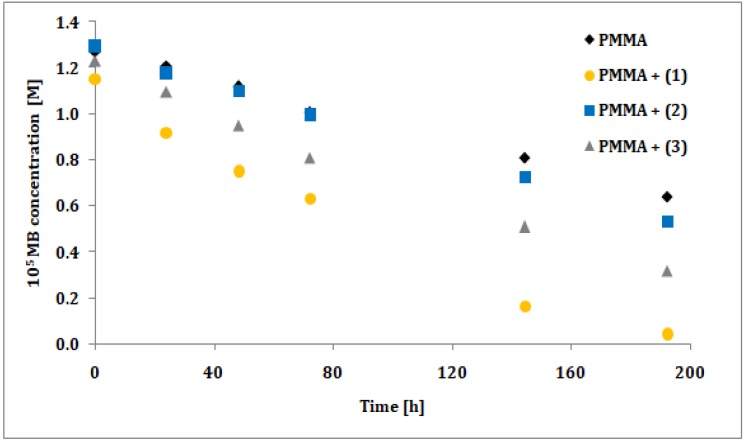
Changes in the concentration of methylene blue (MB) solution under photocatalysis experiment conditions for studied PMMA/{Ti_3_O} (1–3) composite foils.

**Figure 7:**
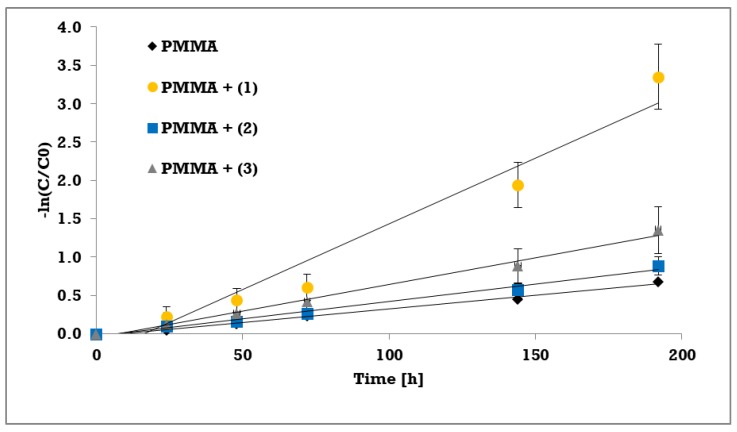
Pseudo-first order fitting of the methylene blue photocatalysis on PMMA foils with selected complexes.

The photocatalytic activity of synthesized trinuclear Ti(IV) oxo-complexes have been estimated basing on the UV photoinduced degradation process of methylene blue (MB), which is widely used as a standard [35,36,37,38,39,40]. Hydrophobic properties of studied compounds caused that photocatalytic experiments were carried out using the polymer/TOCs composites produced by TOCs (**1**–**3**) microcrystals dispersion in the PMMA matrix. As a blind test a pure PMMA foil without any addition was used. The absorption measurements (at wavelength 664 nm) were expressed as methylene blue concentration versus the irradiation time, prior a rate constant calculations. Results of the measurements and pseudo-first order fitting of the methylene blue photocatalysis are presented in Figure 6 and Figure 7, respectively. Apparent rate constants of photodegradation kinetics and decolorization percentage at the end of measurements are presented in Table 6.

## 4. Discussion

The single crystal X-ray diffraction studies of (**2**) and (**3**) allowed to solve their structures as the [Ti_3_O(O^i^Pr)_8_(O_2_CR’)_2_] (R’ = –*p*-PhCl and –*m*-PhNO_2_) clusters. The compounds, which contain a similar type of the titanium-oxo core were also synthesized in the reaction of Ti_3_O(O^i^Pr)_10_ and Ti_3_O(O^i^Pr)_9_(OMe) with benzoic acid at RT in toluene as the solvent [14], and 1:1 reaction of [Ti(OCH_2_Me_3_)_4_]_2_ with such organic acids as HO_2_CH, HO_2_CMe, and HO_2_CH_2_CMe_3_ in toluene [13]. In an environment of the organic acid excess, oxo-complexes of the general formula [Ti_3_O(O^i^Pr)_6_(O_2_C-adamantyl)_4_] (1:1.8, in THF) and [Ti_3_O_2_(O^i^Pr)_3_(O_2_CCF_3_)_5_] (1:2, in ^i^PrOH and CH_3_COOH mixture), can be formed [15]. Contrary to earlier reports oxo-complexes (**2**) and (**3**) were synthesized in the direct reaction of titanium (IV) isopropoxide with 4-chlorobenzoic acid and 3-nitrobenzoic acid, respectively in 4:1 molar ratio at RT in inert atmosphere, using 1:1 THF/^i^PrOH mixture as a solvent. In these conditions were also isolated crystalline powders of (**1**) (9-fluorenecarboxylic acid) and (**4**) (3,3 dimethylacrylic acid), which structures were determined on the basis of IR and Raman spectroscopy.

Analysing the structural data of (**2**) and (**3**), drew attention to the clear impact of the carboxylate group type on the geometry of the {Ti_3_O} bridge, especially on the oxygen atom distance versus plane formed by three titanium atoms. In the case of (**2**) (-*p*-PhCl) this distance is larger than for (**3**) (-*m*-PhNO_2_) and being 0.36Å and 0.24Å, respectively. A similar effect was also noticed for [Ti_4_O_2_(O^i^Bu)_10_(O_2_CR’)_2_] (R’ = –C_13_H_9_, –*m*-PhCl, –*m*-PhNO_2_, –*p*-PhNH_2_) complexes [12]. Due to the weak quality of isolated crystals of (**1**) and (**4**), their structure has been determined basing vibrational spectra (IR and Raman) analysis. Bands, which were found at 1400–1700 cm^−1^ and 900–1050 cm^−1^ proved the presence of coordinated carboxylate and alkoxide groups (Table 4). Moreover between 1400 and 1700 cm^−1^, the presence of bands derived from ν(NO_2_) (**4**) and ν(C=C) ((**1**), (**3**)) was noticed. This fact confirms the coordination of the relevant carboxylate groups in structures of all investigated oxo-clusters. The splitting of ν(Ti-OR) bands (in the range 900–1050 cm^−1^) indicated on the presence of two differently coordinated alkoxide ligands types, i.e. bridging and terminal ones. This is consistent with the structural data of (**2**) and (**3**), which show that {Ti_3_O} core is stabilized by two alkoxide bridges (Figure 1, Table 2). However, the basis of the {Ti_3_O} core identification was the presence of medium or weak bands in IR and Raman spectra of (**1**–**4**) clusters, which can be attributed to the normal vibrations of Ti_3_-(μ_3_-O) bridges (Figure 1). Analysis of data presented in Table 2 indicates that this type of bridge forms a trigonal pyramid belonging to the Cs point group [41], where the oxygen atom forms two Ti-O bonds with the similar lengths and one slightly longer bond. This type of oscillator is represented by six normal vibrations, which are active both the IR and Raman spectra. The use of DFT method allowed on the frequency calculation of normal vibrations for the reference system [Ti_3_O(OMe)_8_(O_2_Me)_2_] and clusters containing studied carboxylate groups (Table 3). Obtained results revealed that the bands derived from stretching and bending vibrations of Ti_3_-(μ_3_-O) bridges should appear at 480–750 cm^−1^ and 340-450 cm^−1^, respectively. The presence of weak/middle bands in above mentioned IR and Raman spectra regions of synthesized (**1**–**4**) compounds may be evidence that the structure of these compounds consists of {Ti_3_O} cores (Figure 2).

The optical properties of (**1**–**4**) oxo-complexes in a broad range of absorption, i.e. between 350 nm and 750 nm, were confirmed by analysis of their UV-Vis-DRS spectra (Table 5). The values of energy gaps change from 1.99 eV (-O_2_CC_13_H_9_ (**1**)) up to 3.23-3.33 eV (-O_2_C-*p*-PhCl (**2**) and -O_2_CC_4_H_7_ (**4**)) dependently to the type of the carboxylate group. It should be noted that above mentioned band gaps determined for trinuclear oxo-complexes are clearly lower compared to those, which were found for [Ti_4_O_2_(O^i^Bu)_10_(O_2_CR’)_2_] (R’ = -C_13_H_9_, -*m*-PhCl, -*m*-PhNO_2_, -*p*-PhNH_2_) that ranged between 2.55 eV (-O_2_CC_13_H_9_) and 3.59 eV (-O_2_C-*m*-PhCl) [12]. The comparison of the band gap determined for trinuclear and tetranuclear clusters exhibited the clear decrease of the band gap energy for oxo-complexes containing fluorenecarboxylate (-O_2_CC_13_H_9_) and *m*-nitrobenzoate (-O_2_C-*m*-PhNO_2_) groups. Moreover, the obtained results showed that the band gap of Ti(IV) oxo-complexes containing -O_2_CPhCl groups decrease up to 3.23 eV–3.59 eV independently to the location of the -Cl substituent in the benzene ring. The lower band gap values of trinuclear oxo-complexes in comparison to the analogous tetranuclear Ti_4_O_2_(O^i^Bu)_10_(O_2_CR’)_2_ complexes may be explained on the basis of structural features and performed DFT calculations. The main factor that sets apart both systems is the presence of five-fold coordinated titanium atom in the structure of trinuclear oxo-complexes, representing slightly disordered trigonal bipyramidal coordination geometry. As it was shown by DFT results, in case of (**1**), (**2**), and (**4**) this particular titanium atom’s d-orbitals hold the highest electronic density of LUMO, which may be reflected in narrowing of the band gap. Similar effect is observed in the case of anatase TiO_2_ crystals with different facets exposed. Crystals dominant with 5 coordinated titanium atoms, i.e. {001} facet exhibit lower bandgap compared to crystals with dominant {101} facet composed of roughly 50% 6-coordinated Ti and 50% 5-coordinated Ti [42]. For discrete structures like presented trinuclear oxo-complex this may have a significant role in the band gap characteristic.

DFT calculations were also carried out in order to determine of the electronic structure of (**1**–**4**) oxo-complexes. Partial density of states (PDOS) plots and calculated highest-occupied molecular orbital (HOMO) and lowest-occupied molecular orbital (LUMO) of studies clusters are presented in Figure 4 and Figure S3 respectively. In the case of (**1**), (**2**), and (**4**) complexes HOMO orbitals are located on corresponding ligands. Main contributors to electron density of these orbitals are π orbitals (phenyl rings for (**1**) and (**2**) and C=C bond for (**4**)) and carbonyl group oxygen of carboxylate ligand (Figure S3). For (**2**) and (**4**), the HOMO orbitals are close to the orbitals of the core, which are mainly composed of core and alkoxides oxygen orbitals, while (**1**) (-O_2_CC_13_H_9_) shows structure characterized by deep penetration of the oxo-titanium core energy gap by ligand orbital (see PDOS plot). The electronic density of LUMO for (**1**), (**2**), and (**4**) is located on titanium atoms of the core. LUMO of (**2**) and (**4**) shows a little contribution of the ligand’s orbitals, while the LUMO of (**1**) is almost solely composed of 3d Ti orbitals of the core. These results indicate that the HOMO–LUMO transition for (**1**), (**2**) and (**4**) involves ligand-to-core charge transfer (LCCT). Compounds (**1**), (**2**) and (**4**) can be described as *n*-type doped semiconductors in regard to the unfunctionalized cluster. The situation is different in case of compound (**3**) where HOMO is located on oxygen atoms of the core and LUMO is composed purely of m-nitrobenzoate ligand, mainly on –NO_2_ group. This complex may be described as *p*-type doped semiconductor with reference to unfunctionalized cluster.

The photocatalytic activity of studied compounds were estimated by methylene blue (MB) UV photoinduced degradation on the surface of composite PMMA foils enriched with TOCs ((**1**–**3**)). Obtained data was processed in terms of MB decolorization percentage after 192 h of experiment, and apparent pseudo-first order rate constant for methylene blue decomposition (Table 6). According to this data, the lowest activity exhibited the PMMA/TOCs (**2**) system, which photocatalytic activity is only slightly different of the reference sample, i.e. pure PMMA foil. Two times faster decomposition was noticed for PMMA/TOCs (**3**), but the most photocatalytic active sample was PMMA/TOCs (1) that elevates photodegradation rate fivefold. The decolorization percentage [24] after 192 h of UV irradiation follows the same trend as kinetic rate constants and changing in the row (**1**) 96% > (**3**) 74% > (**2**) 59% (to reference sample it was 49%). It should be noted that the above-mentioned results change in accordance with the growing values of energy band gaps. Obtained results of photocatalytic activity have been compared to previously studied PMMA-TOCs systems, which contain tetranuclear TOCs with the same carboxylate groups ([Ti_4_O_2_(O^i^Bu)_10_(O_2_CR’)_2_]; R’ = -C_13_H_9_ and -*m*-PhNO_2_) [12]. For the sake of comparison, the rate constants of MB photodegradation on the surface of PMMA-TOCs ({Ti_4_O_2_}) systems were calculated with the same approach as in current study (Table 7).

Analysis of this data revealed that trinuclear Ti(IV) species exhibit better photocatalytic response than tetranuclear Ti(IV) ones with the same carboxylate ligands. Similar to the band gap dependencies, the unsaturated Ti atom may play a paramount role in facilitating the photocatalytic response. In case of TiO_2_, it has been shown that the increased percentage area of {001} facets, rich in fivefold coordinated Ti atoms, is beneficial for both organic contaminations molecules adsorption and retarding charge recombination [42].

## 5. Conclusions

Trinuclear Ti(IV) oxo-complexes [Ti_3_O(O^i^Pr)_8_(O_2_CR’)_2_] (R’ = -C_13_H_9_ (**1**), -*p*-PhCl (**2**), -*m*-PhNO_2_ (**3**), -C_4_H_7_ (**4**)) were isolated as a result of the direct reaction of titanium(IV) isopropoxide with the respective organic acids using 4:1 molar ratio (Ti(O^i^Pr)_4_/HOOCR’) and 1:1 mixture of THF/^i^PrOH as a solvent. The single crystals X-ray diffraction studies proved the formation of trinuclear Ti(IV) oxo-complexes (**2**) and (**3**), while the trinuclear structures of (**1**) and (**4**) (due to the poor quality of the crystals) have been identified basis the analysis of their IR and Raman spectra.

According to diffuse reflectance spectra, the introduction of 9-fluorenecarboxylate ligands into the trinuclear Ti(IV) oxo-complex (**1**) significantly extend the visible absorption range and reduce the band gap in comparison to compounds (**2**) and (**4**), i.e. substituted by 4-chlorobenzoic carboxylate and 3,3-dimethylacrylic carboxylate ligands. In the case of compound (**3**), containing 3-nitrobenzoic carboxylate groups, above mentioned effect is clearly lower than for (**1**).

The results of photocatalytic experiments revealed that activity of synthesized trinuclear oxo-complexes change in the order (**1**) > (**3**) > (**2**). Photocatalytic activity studies of PMMA/TOCs composites (TOCs = [Ti_3_O(O^i^Pr)_8_(O_2_CR’)_2_] and [Ti_4_O_2_(O^i^Bu)_10_(O_2_CR’)_2_], R’ = -C_13_O_9_, -*m*-PhNO_2_) shows clearly that trinuclear Ti(IV) oxo-complexes exhibit the better photocatalytic activity than tetranuclear ones. It suggests that polymer coatings enriched with trinuclear Ti(IV) oxo-complex grains should reveal the appropriate properties as the system, which may be used for photodegradation of the biological/organic pollution.

## Figures and Tables

**Table 1:** The selected crystal data and structure refinements for [Ti_3_O(O^i^Pr)_8_(O_2_CR’)_2_] (R’ = *p-*PhCl (**2)**, *m-*PhNO_2_ (**3**)).

Parameters	(2)	(3)
Empirical formula	C_38_H_64_Cl_2_O_13_Ti_3_	C_38_H_64_N_2_O_17_Ti_3_
Formula weight [g/mol]	943.49	964.61
Temperature [K]	100(2)	100(2)
Wavelength [Å]	0.89429	0.89429
Space group	Triclinic, P -1	Monoclinic, P 2_1_/n
Unit cell dimensions [Å] and angles [°]	A = 10.398(2) b = 11.973(2) c = 20.435(4) α = 77.50(3) β = 80.65(3) γ = 71.71(3)	A = 13.2512(3) B = 22.5559(4) c = 41.475(8) β = 92.632(2) α = γ = 90
Volume [Å3]	2346.1(10)	4655.00(16)
Z, Calculateddensity [Mg/m^3^]	2, 1.336	4, 1.376
Reflectionscollected	33227	63481
Independent reflections	8434 [R(int) = 0.0252]	9408 [R(int) = 0.0340]
Completeness to theta = 32.451°	89.0%	99.0%
Data/restraints/parameters	8434/6/515	9408/0/541
Goodness-of-fit on F2	1.057	1.050
Final R indices [I > 2sigma(I)]	R_1_^a^ = 0.0329, wR_2_^b^ = 0.0843	R_1_^a^ = 0.0293, wR_2_^b^ = 0.0812

^a^ R1 =Σ‖*F_0_*|−|*F_c_*‖/Σ|*F_0_*| ^b^ wR2 = [Σw(*F_0_*^2^−*F_c_*^2^)^2^/Σ(w(*F_0_*^2^)^2^)]^1/2^.

**Table 2:** Selected bond lengths (Å) and angles (°) of [Ti_3_O(O^i^Pr)_8_(O_2_CR’)_2_] (R’ = -*p-*PhCl (**2**), -*m-*PhNO_2_ (**3**).

Parameter		(2)	(3)
Distances [Å]			
Ti-Ti	Ti1-Ti3	3.0787(12)	3.0734(3)
	Ti1-Ti2	3.1455(9)	3.1557(3)
	Ti2-Ti3	3.6297(13)	3.6649(3)
Ti-(µ_3_-O)	Ti1-O5	2.0165(14)	2.0016(9)
	Ti2-O5	2.0012(14)	1.9953(10)
	Ti3-O5	1.8561(15)	1.8513(9)
Ti-(µ_2_-OR)	Ti1-O1	2.0210(15)	2.0177(10)
	Ti1-O6	2.0058(14)	2.0227(10)
	Ti2-O6	2.0097(14)	2.0139(9)
	Ti3-O1	1.9921(14)	1.9806(9)
Ti-OR	Ti1-O11	1.7921(14)	1.7836(10)
	Ti1-O16	1.8019(15)	1.8147(10)
	Ti2-O21	1.8125(15)	1.7834(10)
	Ti2-O26	1.7997(15)	1.8039(10)
	Ti3-O31	1.7875(16)	1.7942(11)
	Ti3-O36	1.7835(16)	1.7505(11)
Ti-O (carb)	Ti1-O51	2.1807(16)	2.1834(10)
	Ti2-O52	2.1242(16)	2.1560(10)
	Ti2-O42	2.0686(14)	2.0705(9)
	Ti3-O41	2.0584(15)	2.0644(10)
O-C (carb)	O41-C42	1.255(2)	1.2605(18)
	O42-C42	1.263(2)	1.2573(17)
	O51-C52	1.262(2)	1.2551(17)
	O52-C52	1.265(2)	1.2631(17)
Angles [deg]			
Ti-(µ_3_-O)-Ti	Ti3-O2-Ti2	140.41(7)	144.61(5)
	Ti3-O2-Ti1	105.23(6)	105.75(5)
	Ti2-O2-Ti1	103.05(6)	104.28(4)
Ti-(µ_2_-OR)-Ti	Ti1-O1-Ti3	100.20(6)	100.47(4)
	Ti1-O6-Ti2	103.13(6)	102.84(4)
O-C-O (carb)	O41-C42-O42	126.12(17)	126.94(12)
	O51-C52-O52	125.69(18)	126.69(13)

**Table 3:** The results of the DFT calculation of Ti-O and Ti-O-Ti frequency modes noticed for {Ti_3_-(µ_3_-O)} bridges of [Ti_3_O(OMe)_8_(O_2_CR’)_2_] clusters. TheO^i^Pr ligands were exchanged on the OMe groups for sake of simplicity. Scaling factor of 1.0007 was applied [29].

Complex	Frequency of Vibrations Involved in ν_s_(Ti_3_-(µ_3_-O)) Bridge (cm^−1^)					
	ν_1_(A‘)	ν_2_(A‘)	ν_5_(A“)	ν_3_(A‘)	ν_4_(A‘)	ν_6_(A“)
[Ti_3_O(OMe)_8_(O_2_CMe)_2_]	730	556	504	446	363	344
[Ti_3_O(OMe)_8_(O_2_CC_13_H_9_)_2_]	722	553	488	446	359	350
[Ti_3_O(OMe)_8_(O_2_C-*p*-PhCl)_2_]	723	554	-	447	355	350
[Ti_3_O(OMe)_8_(O_2_C-*m*-PhNO_2_)_2_]	724	549	-	447	358	340
[Ti_3_O(OMe)_8_(O_2_CC_4_H_7_)_2_]	717	544	-	447	357	340

**Table 4:** frequency of bands assigned to the vibration of coordinated carboxylate groups, isopropoxide ligands, and Ti(IV)-oxo bridges (μ_3_-O) in IR and Raman spectra of studied of (**1**–**4**))complexes (vs = very strong; s = strong; m = medium; w = weak;vw = very weak; b = broad).

Modes	[Ti_3_O(O^i^Pr)_8_(O_2_CC_13_H_9_)_2_] (1)		[Ti_3_O(O^i^Pr)_8_(O_2_CPhCl)_2_] (2)		[Ti_3_O(O^i^Pr)_8_(O_2_CPhNO_2_)_2_] (3)		[Ti_3_O(O^i^Pr)_8_(O_2_CC_4_H_7_)_2_] (4)	
	IR	R	IR	R	IR	R	IR	R
ν(NO_2_)	-	-	-	-	1617 (w) 1600 (m)	1617 (m) 1599 (w)	-	-
ν(C=C)	1610 (s)	1610 (vs)	-	-	-	-	1650 (m)	1650 (s)
ν_as_(COO)	1554 (m)	1552 (m)	1595 (s) 1556 (s)	1595 (s) 1554 (w)	1563 (m) 1536 (s)	1563 (m)	1554 (s)	1552 (m)
ν_s_(COO)	1448 (w)	1448 (m)	1462 (w)	1448 (m)	1438 (m)	1448 (m)	1448 (m)	1449 (s)
ν(C-O) ν(Ti-OR)	1019 (m) 1009 (m)	1023 (s) 978 (w)	1015 (s) 986 (m)	1026 (s)	1014 (vs) 989 (m)	1027 (vs) 1005 (s)	1015 (s) 987 (s)	1027 (vs) 988 (w)
ν_s_(CCC) ν(Ti-OR)	853 (m)	886 (w) 856 (w)	852 (m) 832 (w)	848 (m) 820(w)	854 (m)	856 (w)	867 (w) 849 (m) 833 (w)	856 (w) 837 (w)
δ(CH) (Ph) ν(Ti-O) (μ_3_-O)	740 (m)	747 (m) 725 (w)	770 (m)	725 (w)	765 (w b) 653 (w) 721 (m)	739 (vw) 725 (w)	750 (w) 735 (w)	753 (w) 723 (vw)
δ(CCC) (Ph)	691 (w) 660 (m) 621 (m)	676 (m) 643 (m) 629 (m)	687 (w) 661 (m) 622 (m)	631 (m) 599 (w)	620 (m b)	634 (m) 603 (w)	655 (m)	648 (w) 626 (m)
ν(Ti-O) (μ_3_-O)	575 (w) 545 (vw)	588 (w) 564 (w)	599 (w) 562 (w) 544 (m) 529 (m)	528 (w)	605 (m b) 545 (vw) 529 (w)	535 (vw)	545 (w) 528 (m)	532 (w)
ν(Ti-O) (μ_3_-O)	457 (w) 440 (w)	474 (w) 448 (w)	486 476 (w b)	450 440 (w b)	477 462 (w b) 440	431 (w b)	478 (m b) 457 (w)	458 (w) 426 (m)
	419 (w)	415 (m)	416 (vw)	415 (w)		415 (w)		415 (w)
δ(OTiO) (OR)ν(Ti-O) (μ_3_-O)	-	358 (w) 342(w)	-	332 (m b)	-	345 332 (m b)	-	332 (m b)

**Table 5:** Determined experimentally (DRS) band gaps and theoretically calculated HOMO–LUMO separation gaps (B3LYP/6-31G(d) level of theory). In calculations the O^i^Pr ligands were exchanged with OMe groups.

Complex	Calculated HOMO–LUMO Separation Gap [eV]		Experimental Band Gap [eV]
[Ti_3_O(OMe)_8_(O_2_CC_13_H_9_)_2_]	3.75	[Ti_3_O(O^i^Pr)_8_(O_2_CC_13_H_9_)_2_] (**1**)	1.99
[Ti_3_O(OMe)_8_(O_2_CC_6_H_4_Cl)_2_]	4.24	[Ti_3_O(O^i^Pr)_8_(O_2_CC_6_H_4_Cl)_2_](**2**)	3.23
[Ti_3_O(OMe)_8_(O_2_CC_6_H_4_NO_2_)_2_]	4.20	[Ti_3_O(O^i^Pr)_8_(O_2_CC_6_H_4_NO_2_)_2_ (**3**)	2.84
[Ti_3_O(OMe)_8_(O_2_CC_4_H_7_)_2_]	4.25	[Ti_3_O(O^i^Pr)_8_(O_2_CC_4_H_7_)_2_] (**4**)	3.33

**Table 6:** Rate constants of MB photodegradation on studied materials and MB decolorization percentage.

Sample	MB Decolorization^a^ [%]	10^2^ Rate Constant, [h^−1^]	R^2^
PMMA	49	0.35 ± 0.02	0.991
PMMA + (**1**)	96	1.72 ± 0.19	0.953
PMMA + (**2**)	59	0.45 ± 0.03	0.987
PMMA + (**3**)	74	0.70 ± 0.03	0.990

^a^ MB decolorization at the end of measurements (t = 192 h).

**Table 7:** Comparison of MB photodegradation rate constants for {Ti_3_O} and {Ti_4_O_2_} with the same carboxylate ligands (for tetranuclear Ti (IV) oxo-complexes, rate constant were calculated in accordance with the procedure used in this paper).

TOCs	{Ti_a_O_b_} Core	10^2^ Rate Constant [h^−1^]	R^2^
[Ti_3_O(O^i^Pr)_8_(O_2_CC_13_H_9_)_2_]	{Ti_3_O}	1.72 ± 0.19	0.953
[Ti_4_O_2_(O^i^Bu)_10_(O_2_CC_13_H_9_)_2_]	{Ti_4_O_2_}	1.45 ± 0.11	0.973
[Ti_3_O(O^i^Pr)_8_(O_2_C-*m*-PhNO_2_)_2_]	{Ti_3_O}	0.70 ± 0.03	0.990
[Ti_4_O_2_(O^i^Bu)_10_(O_2_C-*m*-PhNO_2_)_2_]	{Ti_4_O_2_}	0.47 ± 0.03	0.977

## Supplementary Materials

The following are available online at https://www.mdpi.com/1996-1944/12/19/3195/s1. CCDC 1942884 and 1942885 contain the supplementary crystallographic data for (**2**), and (**3**), respectively. This data can be obtained free of charge via http://www.ccdc.cam.ac.uk/conts/retrieving.html, or from the Cambridge Crystallographic Data Centre, 12 Union Road, Cambridge CB2 1EZ, UK; fax: (+44) 1223-336-033; or e-mail: deposit@ccdc.cam.ac.uk. Figure S1: IR spectra of studied trinuclear Ti(IV) oxo-complexes, Figure S2: Raman spectra of studied trinuclear Ti(IV) oxo-complexes, Figure S3: The calculated representation of the HOMO and LUMO orbitals for studied oxo-complexes, Table S1: Atom coordinates for optimized [Ti_3_O(OMe)_8_(O_2_CC_13_H_9_)_2_] structure, Table S2: Atom coordinates for optimized [Ti_3_O(OMe)_8_(O_2_CC_6_H_4_Cl)_2_] structure, Table S3: Atom coordinates for optimized [Ti_3_O(OMe)_8_(O_2_CC_6_H_4_NO_2_)_2_] structure, Table S4: Atom coordinates for optimized [Ti_3_O(OMe)_8_(O_2_CC_4_H_7_)_2_] structure.
