# Supplementary material for: Trinuclear Oxo-Titanium Clusters: Synthesis, Structure, and Photocatalytic Activity
Source: Materials (Basel). 2019 Sep 29;12(19):3195. doi: 10.3390/ma12193195 (PMC6804155; doi:10.3390/ma12193195)
Supplement: Supplementary file 1 [file materials-12-03195-s001.pdf]

materials

Supplementary Materials

Trinuclear Oxo-Titanium Clusters: Synthesis, Structure, and Photocatalytic Activity

Maciej Janek, Tadeusz M. Muzioł and Piotr Piszczek *

Faculty of Chemistry, Nicolaus Copernicus University in Toruń, Gagarina 7, 87-100 Toruń, Poland; maciejjanekk@gmail.com (M.J.); tadeuszmuziol@wp.pl (T.M.M.); piszczek@umk.pl (P.P.)

* Correspondence: piszczek@chem.umk.pl; Tel.: +48-56-611-45-92

Received: 8 August 2019; Accepted: 24 September 2019; Published: date

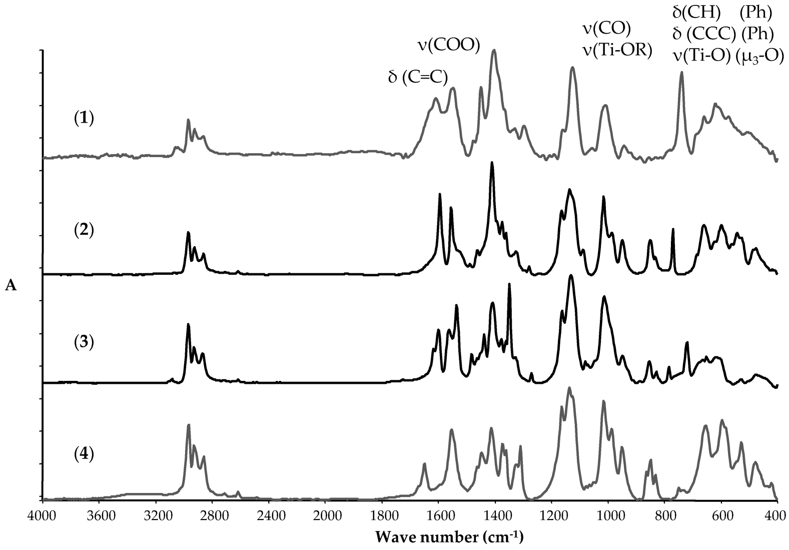

Figure S1. IR spectra of studied trinuclear Ti(IV) oxo-complexes.

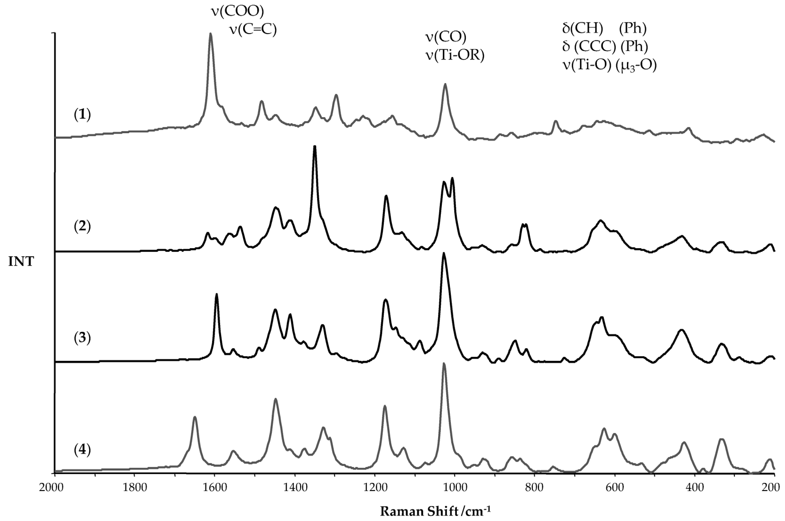

Figure S2. Raman spectra of studied trinuclear Ti(IV) oxo-complexes.

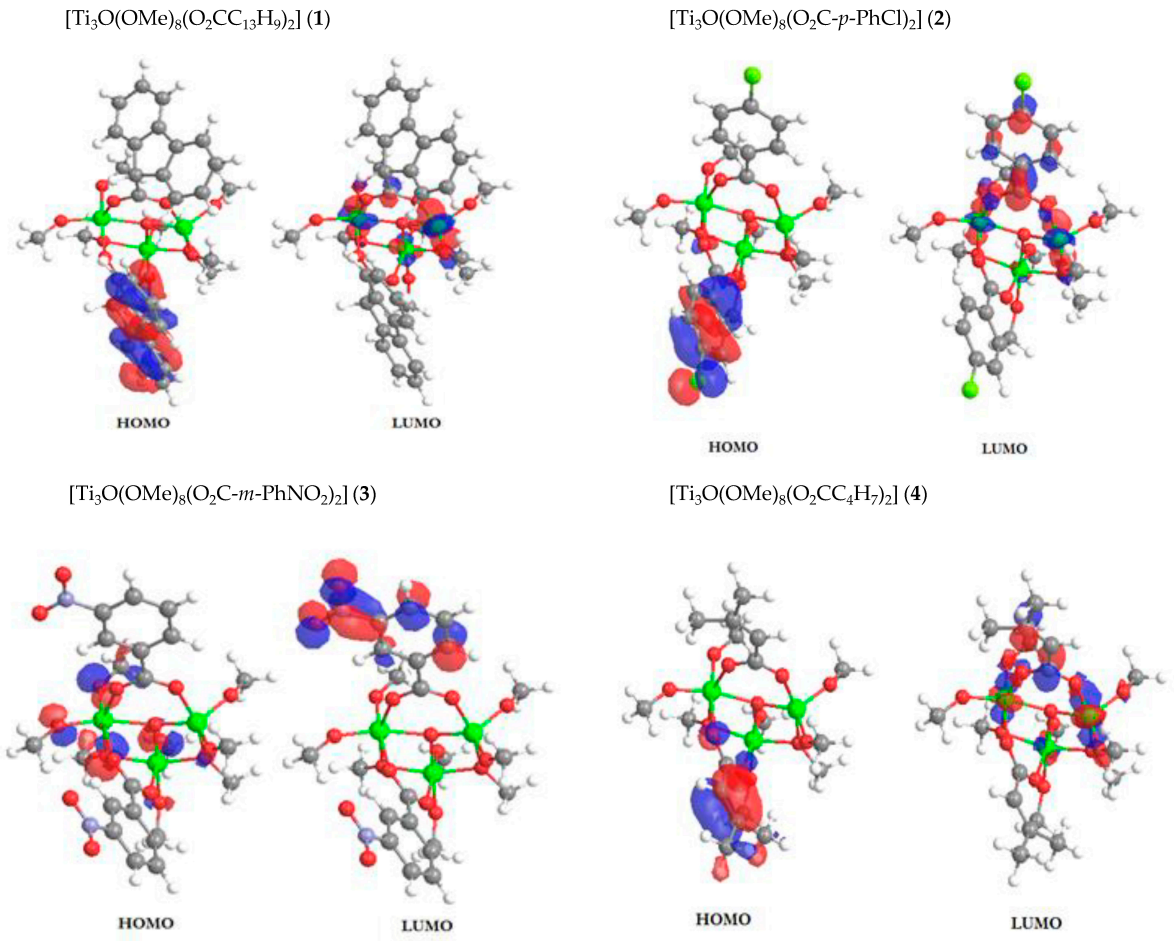

Figure S3. The calculated representation of the HOMO and LUMO orbitals for studied oxo-complexes.

Table S1. Atom coordinates for optimized [$Ti_3O(OMe)_8(O_2CC_{13}H_9)_2$] structure.

Center Number	Atomic Number	Coordinates (Å) X	Y	Z
1	6	5.305746	3.910385	1.606739
2	6	5.941963	2.706236	1.298560
3	6	5.295340	1.798446	0.457262
4	6	4.024050	2.097554	−0.071229
5	6	3.394285	3.298170	0.235118
6	6	4.042978	4.205469	1.080781
7	6	5.712076	0.479153	−0.036499
8	6	4.701771	−0.029884	−0.876304
9	6	3.552363	0.973856	−0.973629
10	6	6.871993	−0.263134	0.193269
11	6	7.005281	−1.513085	−0.416084
12	6	5.995604	−2.016269	−1.243578
13	6	4.831202	−1.274960	−1.480512

14	6	2.230841	0.299472	−0.590640
15	8	1.842604	0.385711	0.609474
16	22	0.233657	−0.297061	1.697679
17	8	1.205906	−0.726767	3.129487
18	6	1.586718	−1.766999	3.995026
19	22	−1.855993	−2.345671	0.675987
20	8	−1.458036	−1.219827	2.319290
21	6	−2.409289	−0.632907	3.196588
22	8	−1.257678	−3.940117	1.280060
23	6	−1.822564	−5.046823	1.934253
24	8	−3.598241	−2.694122	0.976027
25	6	−4.914291	−2.209191	0.990263
26	8	−1.570713	−2.753188	−1.276797
27	6	−1.857509	−4.009694	−1.875874
28	22	0.145131	−1.732198	−1.682535
29	8	−1.139437	−0.035110	−1.761887
30	6	−2.175218	0.246437	−1.082726
31	6	−2.891576	1.538753	−1.526548
32	6	−4.174880	1.914132	−0.812908
33	6	−4.082841	3.223219	−0.299022
34	8	−2.644956	−0.437442	−0.137111
35	8	−0.082244	−1.489722	0.318334
36	8	−0.467544	1.348348	1.766922
37	6	−0.112898	2.634488	2.228451
38	8	1.654923	−0.332355	−1.524704
39	8	1.249545	−3.123786	−1.453380
40	6	1.896144	−3.828669	−0.422354
41	8	0.029774	−1.736603	−3.475049
42	6	−0.524882	−1.060643	−4.569963
43	6	−2.036957	2.791836	−1.349656
44	6	−2.760569	3.767350	−0.634147
45	1	−1.541632	−5.974556	1.417675
46	1	−2.892138	−4.294213	−1.650576
47	1	−2.918145	−4.966024	1.956569
48	1	−1.178211	−4.782545	−1.496668
49	1	−1.735897	−3.927562	−2.961418
50	1	−5.409350	−2.496499	1.928006

51	1	−1.449772	−5.104732	2.966398
52	1	−4.926776	−1.118588	0.893536
53	1	2.010527	−4.880988	−0.714005
54	1	−1.173909	−1.738788	−5.140854
55	1	−5.482596	−2.646122	0.157520
56	1	1.316408	−3.773797	0.506242
57	1	2.896525	−3.407194	−0.253582
58	1	−3.176083	−1.372126	3.451825
59	1	0.275902	−0.711834	−5.235588
60	1	−1.113852	−0.197637	−4.234845
61	1	0.732825	−2.425645	4.201942
62	6	−5.353810	1.189563	−0.680252
63	1	−2.879575	0.235569	2.722236
64	1	2.394005	−2.361982	3.549288
65	1	−1.908782	−0.311072	4.117997
66	6	−0.755197	3.060418	−1.815999
67	1	1.946677	−1.342324	4.940153
68	6	−2.198343	5.021748	−0.383735
69	1	−0.330022	2.728831	3.301151
70	1	0.955615	2.822105	2.064688
71	1	−0.693239	3.384482	1.680416
72	1	7.659644	0.120482	0.836757
73	1	7.903202	−2.100992	−0.244749
74	1	6.115414	−2.990588	−1.709532
75	1	4.042899	−1.667222	−2.117028
76	1	6.923256	2.483261	1.709637
77	1	5.796933	4.626244	2.260581
78	1	3.562430	5.147897	1.329598
79	1	2.410780	3.529755	−0.166138
80	1	3.436531	1.313502	−2.010967
81	6	−5.163862	3.800868	0.370574
82	6	−6.339426	3.062088	0.516287
83	6	−6.436726	1.768585	−0.008893
84	6	−0.911815	5.291776	−0.856326
85	6	−0.196853	4.320565	−1.566715
86	1	−5.435414	0.188105	−1.092501
87	1	−7.362898	1.210669	0.101676

88	1	−7.189508	3.498674	1.034028
89	1	−5.097824	4.810771	0.767504
90	1	−2.753362	5.781457	0.161094
91	1	−0.465720	6.266526	−0.676486
92	1	0.798185	4.550201	−1.939554
93	1	−0.201275	2.302565	−2.360364
94	1	−3.087612	1.390387	−2.599314

Table S2. Atom coordinates for optimized [$Ti_3O(OMe)_8(O_2CC_6H_4Cl)_2$] structure.

Center Number	Atomic Number	Coordinates (Å)		
		X	Y	Z
1	6	0.557811	1.658491	3.124024
2	8	−0.190046	0.690426	2.429010
3	22	−0.051968	−0.995822	1.824621
4	8	0.459535	−2.183646	3.061663
5	6	0.212641	−3.505043	3.480124
6	22	−2.486264	−1.658104	0.028201
7	8	−2.029435	−1.397544	1.996412
8	6	−2.879929	−0.852914	2.993968
9	8	−2.394368	0.525854	−0.020384
10	6	−1.526435	1.272941	−0.560299
11	8	−0.548105	0.868022	−1.262723
12	22	0.012517	−1.111078	−1.827477
13	8	−0.481368	−1.506713	0.107942
14	8	−1.988260	−1.465908	−1.913466
15	6	−2.571656	−2.234572	−2.957172
16	8	−2.617216	−3.458512	−0.001254
17	6	−3.644676	−4.412440	0.078563
18	8	−4.266285	−1.363165	0.063567
19	6	−5.275167	−0.388390	0.051852
20	8	0.196399	−0.450546	−3.486270
21	6	−0.008930	0.694764	−4.268112
22	8	0.506826	−2.805503	−2.117870
23	6	0.708639	−4.006343	−1.416458
24	8	1.860538	−0.570111	−1.092780
25	6	2.424115	−0.470052	0.036282
26	8	1.851263	−0.608914	1.165310

27	6	−1.658528	2.755256	−0.361880
28	6	3.887715	−0.167040	0.047947
29	1	−3.603657	−5.086037	−0.788039
30	1	−3.525319	−5.018077	0.987377
31	1	−4.627191	−3.921220	0.104589
32	1	−6.016104	−0.629080	−0.721947
33	1	−5.789974	−0.365733	1.022224
34	1	−4.851431	0.601849	−0.150948
35	1	−3.663300	−2.144399	−2.909989
36	1	−2.221566	−1.855868	−3.923350
37	1	−2.289393	−3.289911	−2.861986
38	1	−0.627494	0.445752	−5.140928
39	1	−0.507055	1.479643	−3.685752
40	1	0.955287	1.076223	−4.629985
41	1	0.412477	−3.591296	4.555338
42	1	−0.832433	−3.780221	3.286913
43	1	0.867683	−4.205410	2.946111
44	1	0.071873	2.636113	3.021263
45	1	0.626711	1.408812	4.191808
46	1	1.572651	1.724702	2.710080
47	1	−2.454622	−1.049554	3.985539
48	1	−2.981493	0.229985	2.859401
49	1	−3.867108	−1.324001	2.931721
50	1	0.678253	−4.849528	−2.118585
51	1	1.695012	−3.998147	−0.931769
52	1	−0.066172	−4.142542	−0.652756
53	6	4.580693	−0.068246	1.261982
54	6	5.944523	0.209895	1.277501
55	6	6.612549	0.389306	0.065113
56	6	5.942488	0.293608	−1.155634
57	6	4.578820	0.014099	−1.157779
58	1	4.041784	−0.214955	2.191563
59	1	6.486696	0.286437	2.213848
60	17	8.332954	0.741396	0.076207
61	1	6.483008	0.434943	−2.085342
62	1	4.037384	−0.068118	−2.093862
63	6	−0.738183	3.633641	−0.948341

64	6	−0.854623	5.010197	−0.770024
65	6	−1.903374	5.505791	0.005121
66	6	−2.829886	4.650859	0.603240
67	6	−2.700378	3.277000	0.415469
68	1	0.072115	3.225992	−1.542622
69	1	−0.143405	5.692588	−1.223120
70	17	−2.059471	7.241813	0.236136
71	1	−3.636027	5.057737	1.204426
72	1	−3.405841	2.594052	0.875205

Table S3. Atom coordinates for optimized [$Ti_3O(OMe)_8(O_2CC_6H_4NO_2)_2$] structure.

Center Number	Atomic Number	Coordinates (Å)		
		X	Y	Z
1	6	4.310506	−1.256834	−0.193711
2	6	3.384578	−1.385774	0.846308
3	6	3.829421	−1.662825	2.146563
4	6	5.191321	−1.809478	2.410727
5	6	6.123115	−1.681496	1.382413
6	6	5.661765	−1.406896	0.095280
7	6	1.920027	−1.232593	0.559214
8	8	1.125400	−1.340283	1.546439
9	22	−0.914148	−1.190495	1.822108
10	8	−1.106800	−1.324723	−0.003062
11	22	−3.017594	−0.849168	−0.421532
12	8	−3.647949	−2.502241	−0.763334
13	6	−4.896837	−3.093134	−1.018430
14	1	7.186815	−1.789316	1.555414
15	8	1.591326	−1.013270	−0.640725
16	22	−0.147600	−0.874702	−1.747064
17	8	−0.095314	−2.591921	−2.240684
18	6	−0.374918	−3.869745	−1.726976
19	8	−0.226766	1.106839	−0.933154
20	6	−1.170526	1.698484	−0.327147
21	8	−2.299853	1.199843	−0.053820
22	6	−0.919678	3.121568	0.098685
23	6	−1.898193	3.833904	0.804999
24	6	−1.671710	5.153506	1.198323

25	6	−0.465131	5.779658	0.892298
26	6	0.499324	5.055664	0.191923
27	6	0.295542	3.739513	−0.209282
28	1	−0.258822	6.802551	1.182159
29	8	−2.105196	−0.581977	−2.191841
30	6	−2.666345	−0.951685	−3.445228
31	8	0.549269	−0.108900	−3.204308
32	6	0.909781	1.114027	−3.791183
33	8	−4.599634	−0.012267	−0.644352
34	6	−5.256898	1.226641	−0.690072
35	8	−2.912794	−0.977524	1.607504
36	6	−3.766809	−0.335943	2.543030
37	8	−0.685753	0.372959	2.682256
38	6	0.107183	0.889092	3.725629
39	8	−1.004429	−2.606761	2.907111
40	6	−1.622592	−3.859648	3.080684
41	1	−4.860049	−3.661966	−1.956916
42	1	−5.156018	−3.787269	−0.207474
43	1	−5.678862	−2.325098	−1.094018
44	1	−5.878818	1.284783	−1.592937
45	1	−5.912617	1.338579	0.184280
46	1	−4.531203	2.048004	−0.698599
47	1	−3.681622	−0.546178	−3.523727
48	1	−2.050146	−0.539017	−4.250816
49	1	−2.701346	−2.043260	−3.544589
50	1	0.523793	1.160185	−4.817794
51	1	0.505946	1.954624	−3.213959
52	1	2.003166	1.199104	−3.828878
53	1	−1.717140	−4.074592	4.152144
54	1	−2.621685	−3.863043	2.626173
55	1	−1.019020	−4.650928	2.618042
56	1	−0.122051	1.952582	3.863385
57	1	−0.087576	0.361422	4.669482
58	1	1.172162	0.789344	3.478086
59	1	−3.684944	−0.834254	3.516702
60	1	−3.484576	0.717017	2.656576
61	1	−4.804493	−0.401426	2.198544

62	1	−0.588006	−4.558215	−2.554618
63	1	0.497465	−4.251887	−1.178605
64	1	−1.238762	−3.835298	−1.052619
65	1	3.096664	−1.763558	2.939662
66	1	5.529707	−2.024673	3.419617
67	7	6.642914	−1.273493	−0.997641
68	1	3.983301	−1.045435	−1.203246
69	1	1.065226	3.203045	−0.748101
70	7	1.779503	5.709463	−0.132942
71	1	−2.436096	5.697685	1.744945
72	1	−2.831930	3.336372	1.041668
73	8	1.937652	6.871518	0.242049
74	8	2.613436	5.056793	−0.758352
75	8	6.214349	−1.029175	−2.123398
76	8	7.832126	−1.416619	−0.714264

Table S4. Atom coordinates for optimized [$Ti_3O(OMe)_8(O_2CC_4H_7)_2$] structure.

Center Number	Atomic Number	Coordinates (Å)		
		X	Y	Z
1	6	1.061352	−3.721744	−0.238508
2	8	1.165230	−2.722547	−1.218725
3	22	0.851437	−0.970340	−1.438690
4	8	0.440984	1.099638	−1.490508
5	6	−0.601982	1.755242	−1.156333
6	6	−0.525507	3.217124	−1.412549
7	8	2.535387	−0.414760	−0.409889
8	6	2.848929	−0.022991	0.754353
9	8	2.017135	0.131717	1.716178
10	22	0.012916	−0.027352	1.997219
11	8	−0.092841	1.751523	2.290378
12	6	0.657048	2.736272	2.958504
13	8	0.110682	−1.000187	3.496987
14	6	−0.334626	−2.214025	4.051491
15	8	−0.107968	−0.851542	0.353347
16	22	−2.042251	−0.853763	−0.173787
17	8	−1.647804	1.231602	−0.662837
18	8	−1.990118	−0.218466	1.759271

19	6	−2.979208	0.488468	2.487542
20	8	1.486047	−0.766647	−3.110413
21	6	1.588440	0.168982	−4.148956
22	8	−1.115986	−1.182996	−1.933449
23	6	−1.543428	−2.154368	−2.877683
24	8	−2.423665	−2.595394	0.132466
25	6	−3.572847	−3.400476	0.132314
26	8	−3.734599	−0.408078	−0.630829
27	6	−4.554572	0.624634	−1.109409
28	1	1.037580	−4.708576	−0.719639
29	1	0.411272	−3.003760	3.892466
30	1	1.933660	−3.687330	0.429849
31	1	−0.484318	−2.090596	5.131608
32	1	0.149208	−3.589040	0.355466
33	1	−1.282463	−2.525593	3.593425
34	1	−3.735493	−3.825693	1.132554
35	1	−3.454065	−4.232631	−0.575300
36	1	−1.407803	−3.166936	−2.478926
37	1	−4.456357	−2.812197	−0.152416
38	1	−0.953545	−2.050258	−3.794945
39	1	−2.937290	0.197732	3.545184
40	1	1.728923	2.601585	2.762020
41	1	−2.602817	−1.995330	−3.111922
42	1	0.489219	2.690005	4.043908
43	1	−3.972489	0.247692	2.093318
44	1	2.626916	0.219205	−4.503016
45	1	0.954317	−0.134675	−4.993642
46	1	−2.807115	1.568791	2.413287
47	1	1.276668	1.163680	−3.807018
48	1	0.356520	3.729412	2.601101
49	1	−5.243207	0.958138	−0.320354
50	1	−5.155418	0.261358	−1.954060
51	1	−3.948693	1.477268	−1.435955
52	6	−1.302008	4.203151	−0.921749
53	1	0.308052	3.486553	−2.056461
54	6	−1.026960	5.634090	−1.315161
55	6	−2.456225	4.020672	0.028180

56	1	−2.384510	4.746865	0.848731
57	1	−3.405870	4.227117	−0.486341
58	1	−2.494753	3.011951	0.433900
59	1	−1.920155	6.089610	−1.764952
60	1	−0.782227	6.240499	−0.432254
61	1	−0.202312	5.717251	−2.029026
62	6	4.254186	0.280870	1.097890
63	6	5.340083	0.287928	0.295388
64	1	4.380323	0.536265	2.146448
65	6	6.684847	0.637270	0.883802
66	6	5.364355	−0.025150	−1.176784
67	1	7.401772	−0.177802	0.715617
68	1	7.103296	1.524267	0.388974
69	1	6.631803	0.834224	1.958269
70	1	5.863917	0.788320	−1.720249
71	1	5.967896	−0.927101	−1.351266
72	1	4.372181	−0.184877	−1.592493
